# Too much of a good thing: OTC calcium carbonate overdose causing milk-alkali syndrome in a psychiatric patient

**DOI:** 10.1186/s12991-025-00615-4

**Published:** 2025-12-24

**Authors:** Alexandra She, Sangay Bhutia, Francis E Jenney, Jr

**Affiliations:** 1 Philadelphia College of Osteopathic Medicine GA Campus, Suwanee, GA USA; 2 Piedmont Columbus Family Medicine , Columbus, GA USA

**Keywords:** Milk-alkali syndrome, Hypercalcemia, Bipolar disorder, Altered mental status, Psychiatric comorbidity, Case report.

## Abstract

**Background:**

In the United States, milk-alkali syndrome or calcium-alkali syndrome is the third most common cause of hypercalcemia, after primary hyperparathyroidism and malignancy. Clinically, patients present with a triad of hypercalcemia, acute kidney injury, and metabolic alkalosis. Classically, it has been associated with excessive intake of calcium and absorbable alkali, and recently it has become more common in postmenopausal females who ingest calcium supplements for osteoporosis.

**Case presentation:**

Here, we present the case of a 72-year-old Caucasian female with a longstanding diagnosis of bipolar I disorder who presented to the Emergency Department (ED) with a sudden onset of generalized weakness, multiple falls, and altered mental status over the course of several days. On admission, she had elevated serum calcium, acute kidney injury, and metabolic alkalosis.

**Conclusions:**

This case underscores the importance of thorough medical and psychiatric assessment in patients presenting with altered mental status, particularly in those with a history of overdosing. It also highlights the need for close collaboration between psychiatry and internal medicine in the management of metabolic disturbances that may mimic or exacerbate psychiatric symptoms. A stepwise approach to the diagnosis and treatment of hypercalcemia is reviewed, with particular attention to considerations unique to psychiatric populations.

## Background

**Fig. 1:**
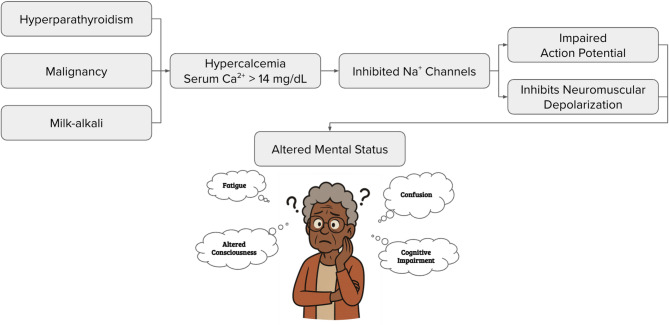
Proposed mechanism of hypercalcemia-induced altered mental status [4]

Milk-alkali syndrome results from excessive calcium carbonate intake, commonly from supplements for osteoporosis or acid reflux [1–3]. The proposed mechanism of hypercalcemia leading to altered mental status involves the inhibition of sodium channels, which leads to dampened neuronal and muscular excitability (Fig. 1) [4]. This results in fatigue, cognitive decline, muscle weakness, hypotonia, and slowed reflexes. Cardiac effects of hypercalcemia include arrhythmias such as bradycardia, prolonged PR interval, shortened QT, and widened QRS complexes [5]. Neurologically, patients may exhibit confusion, poor concentration, and irritability. Calcium levels above 14 mg/dL can lead to encephalopathy, while levels exceeding 15 mg/dL constitute a medical emergency due to the risk of life-threatening complications [6]. 

## Case presentation

In October 2024, a 72-year-old Caucasian female with a complex medical history of anemia, type 2 diabetes mellitus, duodenal and gastric ulcers, and an overactive bladder, as well as a psychiatric history notable for bipolar I disorder, generalized anxiety disorder (GAD), and insomnia, presented to the ED via ambulance. The chief complaints were altered mental status of one day’s duration and multiple falls over the preceding six to seven days. Upon arrival, the patient was obtunded and unable to provide a coherent history. Collateral information from her husband revealed a recent rapid decline regarding baseline ambulation, characterized by six falls in the week prior, new-onset unsteadiness, and generalized weakness, described as episodes where her limbs “simply give out” without support. He denied any baseline gait abnormalities or environmental factors that might explain the falls. Although a walker was available at home, it had only been used regularly in the days immediately preceding admission, coinciding with her functional deterioration. Additionally, the patient required assistance with activities of daily living (ADLs), including bathing and medication management, provided by a home caregiver who recognized her altered mental status and initiated emergency medical services, suspecting a transient ischemic attack.

The husband reported two psychiatric hospitalizations in 2024 for manic episodes, the most recent episode being associated with an intentional overdose of unknown medications. The patient’s diagnosis of bipolar I disorder dates to approximately 2004–2005, following the death of her father. The patient’s family history was notable only for a maternal seizure disorder. The husband denied that the patient had any history of tobacco, alcohol, or illicit drug use. The patient lived at home with her husband, who traveled frequently for work and managed household finances.

Past pharmacological management included lithium and ziprasidone, which have both been discontinued. Her current medications included quetiapine 100 mg nightly and lamotrigine 100 mg twice daily for bipolar I disorder; trazodone 50 mg nightly and mirtazapine 7.5 mg nightly for insomnia; hydroxyzine 25 mg twice daily for anxiety; tolterodine 4 mg extended-release as needed for overactive bladder; polyethylene glycol, docusate sodium, and linaclotide for constipation; fluconazole for yeast infection; and pantoprazole 40 mg twice daily for gastric and duodenal ulcer disease.

On physical examination, the patient was disoriented to person, place, time, and situation. Her speech was notably slurred, punctuated by occasional incomprehensible sounds. At one point, she fixated briefly on her fingernails and uttered the word “purple.” Despite her altered mental status, she did not exhibit signs of acute distress. Respiratory examination revealed clear bilateral lung fields without wheezing. Cardiac auscultation demonstrated normal S1 and S2 heart sounds with a regular rate and rhythm. Her abdomen was soft and nondistended, with mild tenderness on deep palpation in the bilateral lower quadrants. There was no peripheral edema. Neurologically, motor testing showed generalized weakness in all four extremities, with muscle strength graded at 3/5 bilaterally.

### Laboratory and imaging studies

Initial laboratory investigations included a complete blood count (CBC, Table 1), comprehensive metabolic panel (CMP, Table 2), ethanol level, vitamin D 25-hydroxy (Table 3), vitamin D 1,25-dihyrdroxy (Table 4), thyroid-stimulating hormone (TSH), phosphorus, ionized calcium (Table 5), urinalysis (Table 7), urine drug screen, ammonia, vitamin B12 levels, chest radiograph, electrocardiogram (EKG), non-contrast head computed tomography (CT), and a viral panel screening for COVID-19, influenza A and B, and respiratory syncytial virus (RSV). Laboratory values for ethanol, TSH, phosphorus, urine drug screen, ammonia, B12, and viral panel were all within normal limits or negative. Only abnormal laboratory findings are detailed below.

**Table 1:** CBC

CBC	Result	Reference range
WBC	13.10 * 10^3^ (H)	4.00–10.50 * 10^3^/µL
Neutrophils Relative	84.5 (H)	30.0–70.0%
Neutrophils Absolute	11.0 (H)	1.8–8.0 * 10^3^/µL
Lymphocytes Relative	15.6% (L)	24.0–44.0%

**Table 2:** CMP

CMP	Result	Reference range
Sodium (Na^+^)	138	136–145 mmol/L
Potassium (K^+^)	2.5 (L)	3.5–5.1 mmol/L
Chloride (Cl^−^)	91 (L)	98–107 mmol/L
Magnesium (Mg^2+^)	1.9 mg/dL	1.9–2.7 mg/dL
Phosphate (PO_4_^3−^)	2.5 mg/dL	2.4–4.5 mg/dL
CO_2_	35 (H)	21–31 mmol/L
Glucose	127 (H)	74–109 mg/dL
BUN	62 (H)	7–25 mg/dL
Creatinine	3.09 (H)	0.5–1.2 mg/dL
Calcium	> 18.0 (H)	8.6–10.3 mg/dL
AST	79 (H)	< 39 U/L
Alkaline Phosphatase	133 (H)	34–103 U/L
GFR CKD-EPI	15 (L)	>= 60 mL/min/1.73m^2^

An arterial blood gas (ABG) was not completed, but the metabolic alkalosis was supported by the CMP, which showed a low chloride level of 91 mmol/L and an elevated bicarbonate level of 35 mmol/L (see Table 2).

**Table 3:** Vitamin D 25-hydroxy

Vitamin D 25-hydroxy	Reference range
26.7 (L)	30.0–100.0 ng/mL

**Table 4:** Vitamin D 1,25-dihyrdroxy

Vitamin D 1,25-dihydroxy	Reference range
< 8 (L)	18–72 pg/mL

**Table 5:** Ionized calcium

Ionized calcium	Reference range
2.24 (H)	1.12–1.32 mmol/L

**Table 6:** Urinalysis

Urinalysis (UA)	Result	Reference range
Protein, UA	2+ (> 100 mg/dL) (H)	Negative
Blood, UA	2+ (H)	Negative

Of note, no urine casts were reported

(L): low; (H): high

The EKG was benign and demonstrated normal sinus rhythm, a prolonged QT interval, and a subtle QRS duration change compared to a prior EKG from April 2024. The EKG did not show the presence of Osborn (J) waves. Cardiac enzymes and echocardiography were not performed due to the benign EKG and the lack of chest pain on presentation. Additionally, the chest radiograph showed no acute cardiopulmonary process. The head CT without contrast did not identify any acute intracranial abnormalities.

## Therapeutic interventions

Severe hypercalcemia constitutes a medical emergency due to its potential life-threatening complications, necessitating prompt intervention even before the underlying etiology is fully elucidated. Given the patient’s critically elevated serum calcium level exceeding 18 mg/dL and an ionized calcium concentration of 2.24 mmol/L, immediate treatment was initiated. This included aggressive intravenous hydration with 0.9% normal saline at 1,000 mL and 2,000 mL bolus IV, a continuous infusion of 0.9% normal saline at a rate of 125 mL/h, administration of calcitonin 280 units subcutaneously every 12 h, pamidronate 90 mg/10 mL in 0.9% normal saline given IV given once, and zoledronic acid 4 mg in 100 mL of 0.9% normal saline given IV.

Additionally, a comprehensive list of all the medications administered during the 10 day hospitalization is shown in Table 7.

**Table 7:** Comprehensive list of medications given during the hospitalization

Medication	Dosage	Route	Frequency/Timing	Duration**
calcitonin	280 units (4 units/kg)*	SubQ	q12h	3 days
calcitriol	0.25 mcg	PO	daily	2 days
Docusate sodium	100 mg	PO	BID	2 days
ergocalciferol	50,000 units	PO	weekly	Given once
fluoxetine	40 mg	PO	Daily with breakfast	10 days
Insulin lispro	1–6 units	SubQ	TID before meals	5 days
lamotrigine	100 mg	PO	BID/qhs	10 days
Magnesium sulfate	3 g in NaCl 0.9%	IV	once	1 day
mirtazapine	7.5 mg	PO	qhs	10 days
Pantoprazole	40 mg	PO	BID	10 days
Potassium chloride	10 mEq in 100 mL	IV	q2h	1 day
quetiapine	100 mg	PO	daily	10 days
senna-docusate	8.6–50 mg	PO	qhs	3 days
Trazodone	50 mg	PO	qhs	10 days
Zoledronic acid	4 mg in NaCl 0.9% 100 mL	IV	once	1 day

* patient weighed 68.9 kg; ** patient was hospitalized for a total of 10 days

## Discussion and conclusions

Serum parathyroid hormone (PTH) was ordered to evaluate for primary hyperparathyroidism as a potential cause. A comprehensive medication review was conducted to identify possible pharmacologic contributors. Lithium, known to induce hypercalcemia by either exacerbating pre-existing hyperparathyroidism or causing direct parathyroid gland injury, elevates calcium levels in approximately 20% of users [7]. Notably, cases of lithium-induced hypercalcemia with normal PTH levels have also been documented [7]. However, the patient’s husband explicitly denied any current lithium use, thereby excluding this etiology. High-dose fluconazole has been implicated in rare instances of severe hypercalcemia secondary to exacerbation of hyperparathyroidism, particularly among patients treated for coccidioidomycosis [8]. This complication is very rare and typically occurs at substantially higher doses than those relevant to this patient. Hence, this cause was deemed improbable.

Since the PTH levels (Table 8) came back normal, further evaluation focused on malignancy as a potential source. A non-contrast panoramic CT scan revealed no evidence of metastatic disease. Subsequent brain MRI demonstrated no acute intracranial pathology or abnormal contrast enhancement. Additionally, parathyroid hormone-related protein (PTHrP) levels were low, effectively excluding hypercalcemia of malignancy [9]. 

**Table 8:** Markers of hyperparathyroidism and malignancy

Markers	Result	Reference range
PTH	25.47	12.00–88.00 pg/mL
PTH-rP	7 (L)	11–20 pg/mL

(L): low; (H): high

Multiple myeloma, the second most common hematologic malignancy in the United States, can present with hypercalcemia [10, 11]. However, a review of the patient’s medical records revealed a previously negative workup for this condition. The refractory nature of this patient’s peptic ulcer disease in conjunction with hypercalcemia brought up the possibility of multiple neuroendocrine neoplasia type 1 (MEN 1) syndrome [12]. However, given that prolactin studies were normal and the PTH was not elevated, MEN 1 syndrome was ruled out. Although rare, pheochromocytoma has been associated with hypercalcemia and ectopic calcitonin secretion in isolated case reports [13]. Nonetheless, the patient did not exhibit classic symptoms of pheochromocytoma such as diaphoresis, tachycardia, or headaches, and serum metanephrine levels were within normal range, making this diagnosis unlikely. Sarcoidosis was also considered given its potential to cause hypercalcemia [14]. However, the typical laboratory markers, such as elevated angiotensin-converting enzyme (ACE) and calcitriol level, were absent. Furthermore, the characteristic bilateral hilar lymphadenopathy on chest imaging, a hallmark of sarcoidosis, was not observed. Lastly, the possibility of hypogammaglobulinemia was evaluated due to findings on protein electrophoresis; however, this was attributed to acute inflammatory processes rather than a primary immunodeficiency [15]. 

**Fig. 2:**
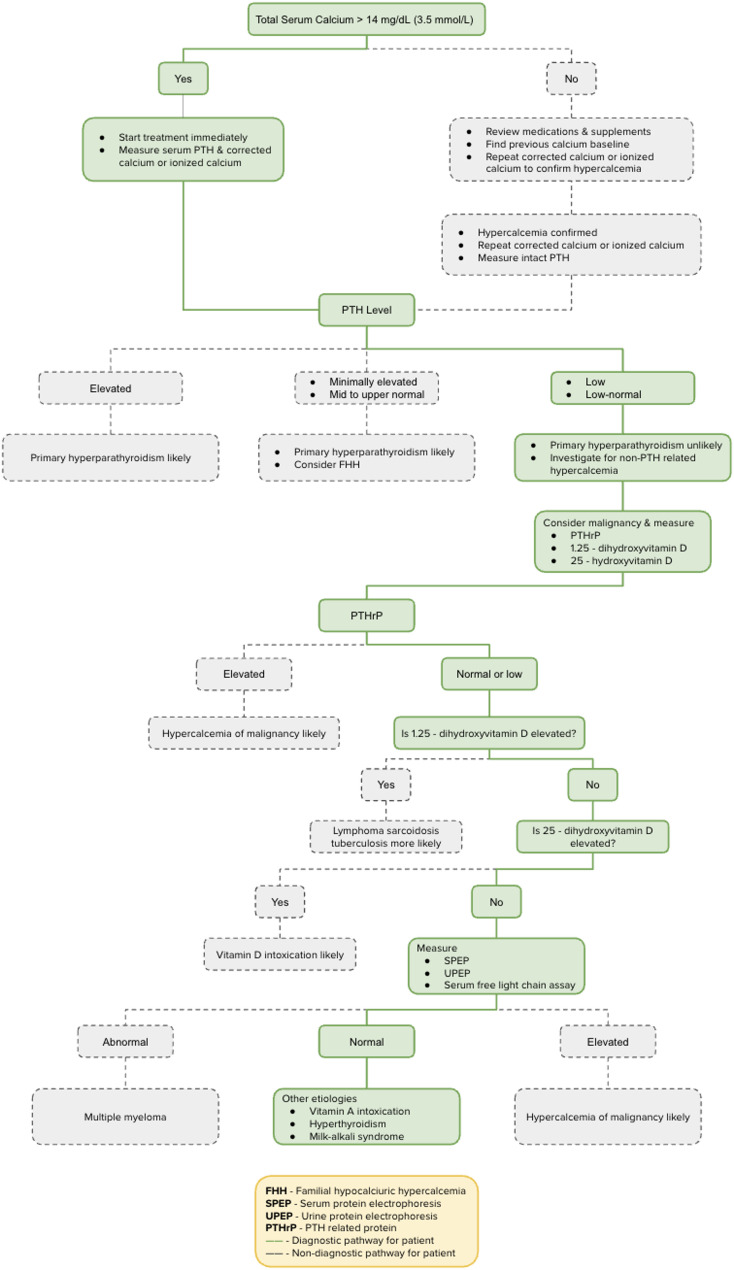
Stepwise diagnostic approach to hypercalcemia. (adapted from UpToDate) [16]

A stepwise diagnostic approach for the patient at hand is presented in Fig. 2.

**Fig. 3:**
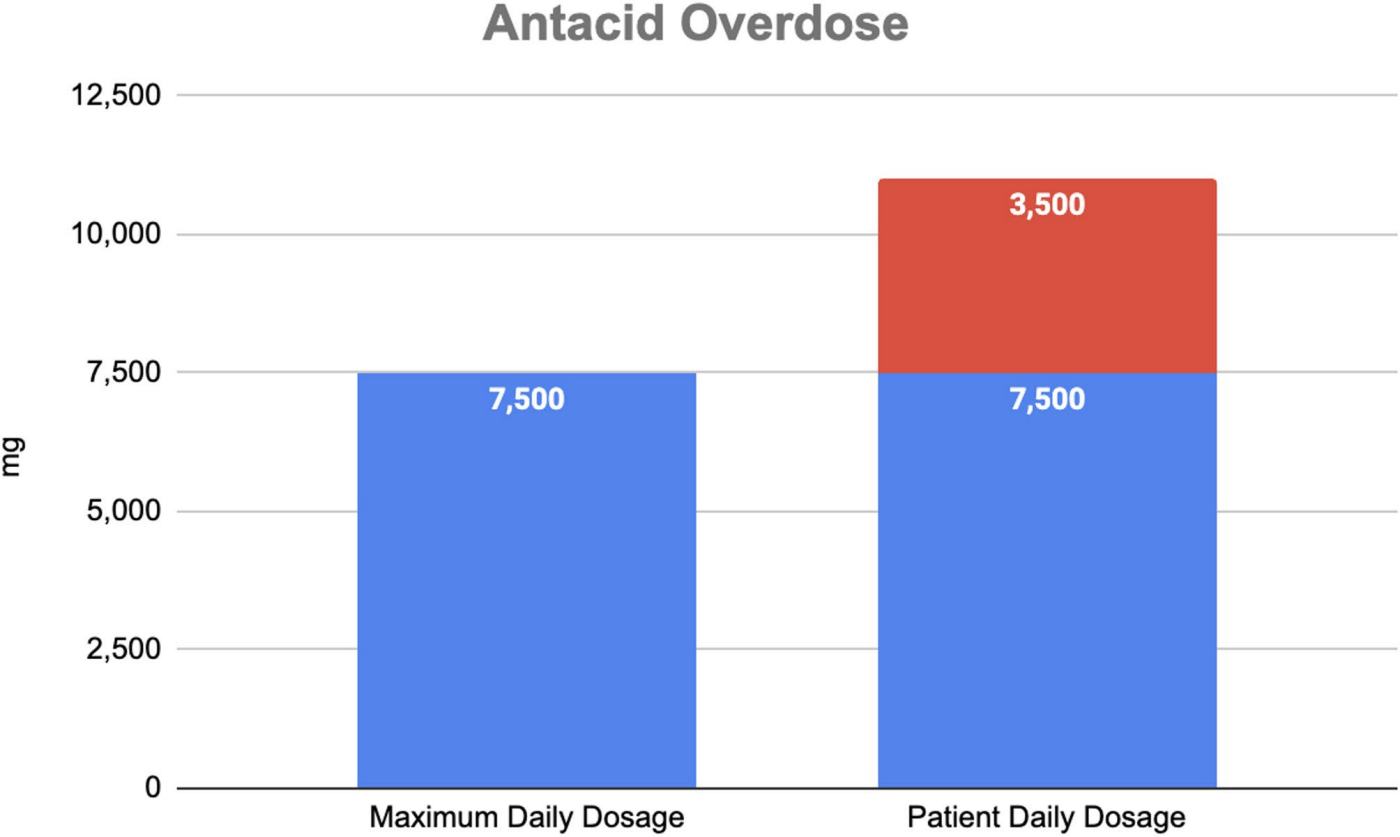
Antacid overdose

Given the limitations in obtaining a reliable history during the patient’s initial ED presentation, a follow-up interview was conducted with the patient and her caregivers to explore alternative etiologies, particularly the possibility of excessive calcium intake. Although the patient’s mental status had improved, she continued to exhibit disorientation to time and place. When directly asked about antacid use, she denied any recent consumption. However, separate interviews with her husband and home caregiver revealed a critical piece of information: the patient had been consuming large quantities of over-the-counter calcium carbonate (Tums) in the two weeks preceding her hospitalization. According to her caregiver, the patient had independently purchased two large bottles of Tums and reportedly consumed one full bottle of 160 to 170 chewable tablets within roughly one week. Each tablet contains 500 mg of elemental calcium, equating to an estimated average daily intake of 11,000 mg. The recommended dosage for symptomatic heartburn is 1,500 to 3,000 mg per dose, with a maximum daily limit of 7,500 mg. [17] Thus, the patient had significantly exceeded the safe daily threshold for calcium intake. This newly acquired information led to the clear diagnosis of milk-alkali syndrome. A bar graph depicting the overdose of antacids is shown in Fig. 3.

The likely etiology of hypokalemia is renal potassium wasting secondary to hypercalcemia-induced nephrogenic diabetes insipidus and resultant polyuria. Hypercalcemia impairs the renal concentrating ability by interfering with the responsiveness of the collecting ductal antidiuretic hormone (ADH), leading to polyuria, volume depletion, and increased distal sodium delivery. These changes collectively enhance renal potassium excretion. Additionally, in milk-alkali syndrome, metabolic alkalosis arises from excessive alkali intake and subsequent bicarbonate retention. This alkalosis further promotes renal potassium loss by increasing potassium secretion in the distal nephron.

Calcium gluconate 2 g in NaCl 0.9% given IV was used on the 9th day of hospitalization. This medication was administered because the patient’s serum calcium levels began to drop, and the ionized calcium level decreased to 0.9 mg/dL. The patient’s low calcium levels were likely secondary to the aggressive treatment of hypercalcemia. For these reasons, the patient was supplemented with calcium gluconate.

This case punctuates the importance of thorough and systematic history taking in the ED, particularly in patients with complex psychiatric backgrounds, medical histories, or those who are too obtunded to adequately communicate. In this instance, the initial omission of over-the-counter calcium carbonate use delayed the identification of the true etiology of the patient’s hypercalcemia, demonstrating how even non-prescription substances can have significant clinical consequences when not disclosed. Therefore, obtaining collateral information from the next of kin and caregiver proved to be critical to delivering timely and adequate medical care to the patient at hand. Additionally, this case serves as a reminder of how crucial patient education can be, especially regarding the risks associated with over-the-counter medications, such as antacids, which are often perceived as harmless. In psychiatric patients, the potential for unintentional overdose can be heightened due to prior overdose attempts, impaired judgment, or difficulty understanding medication guidelines. While the patient’s prior overdose was intentional, this incident demonstrates that even inadvertent misuse of common medications can lead to severe and potentially fatal consequences. Comprehensive psychiatric evaluation remains essential not only for managing mental illness but also for anticipating behaviors that may predispose patients to medical emergencies, regardless of intent.

## Data Availability

No datasets were generated or analysed during the current study.

## Abbreviations

ABG: Arterial blood gas
ADH: Anti-diuretic hormone
ADLs: Activities of daily living
ACE: Angiotensin-converting enzyme
AST: Aspartate Aminotransferase
BUN: Blood urea nitrogen
CBC: Complete blood count
CMP: Comprehensive metabolic panel
CO₂: Carbon dioxide
CT: Computed tomography
EKG: Electrocardiogram
ED: Emergency department
GAD: Generalized anxiety disorder
GFR: Glomerular filtration rate
CKD-EPI: Chronic kidney disease epidemiology collaboration
MEN 1: Multiple endocrine neoplasia type 1
MRI: Magnetic resonance imaging
PTH: Parathyroid hormone
PTHrP: Parathyroid hormone-related protein
QRS: Ventricular depolarization complex (on EKG)
QT: Ventricular depolarization to repolarization interval (on EKG)
RSV: Respiratory syncytial virus
S1/S2: First / Second Heart Sounds
ST: ST Segment (on EKG)
TSH: Thyroid-stimulating hormone
UA: Urinalysis
WBC: White blood cell count
mg: Milligram
mL: Milliliter
ng/mL: Nanograms per milliliter
pg/mL: Picograms per milliliter
mmol/L: Millimoles per liter
mg/dL: Milligrams per deciliter
U/L: Units per Liter
COVID-19: Coronavirus disease 2019
RSV: Respiratory syncytial virus
H: High (above reference range)
L: Low (below reference range)
